# Novel protective effects of pulsed electromagnetic field ischemia/reperfusion injury rats

**DOI:** 10.1042/BSR20160082

**Published:** 2016-12-05

**Authors:** Fenfen Ma, Wenwen Li, Xinghui Li, Ba Hieu Tran, Rinkiko Suguro, Ruijuan Guan, Cuilan Hou, Huijuan Wang, Aijie Zhang, Yichun Zhu, YiZhun Zhu

**Affiliations:** * Department of Pharmacy, Shanghai Pudong Hospital, Fudan University, Shanghai 201399, China; † Shanghai Institute of Immunology & Department of Immunobiology and Microbiology, Shanghai Jiao Tong University School of Medicine, Shanghai 200025, China; ‡ Shanghai Key Laboratory of Bioactive Small Molecules and Research Center on Aging and Medicine, Department of Physiology and Pathophysiology, Shanghai Medical College, Fudan University, Shanghai 200032, China; § Department of Pharmacology, School of Pharmacy, Fudan University, Shanghai 201203, China; ∥ Longhua Hospital, Shanghai University of Tradition Chinese Medicine, Shanghai 201203,China; ¶ Department of Pharmacology, Yong Loo Lin School of Medicine, National University of Singapore 119228, Singapore

**Keywords:** apoptosis, Bax, B-cell lymphoma 2 (Bcl-2), ischaemia/reperfusion (I/R) injury, pulsed electromagnetic field (PEMF)

## Abstract

Extracorporeal pulsed electromagnetic field (PEMF) has shown the ability to regenerate tissue by promoting cell proliferation. In the present study, we investigated for the first time whether PEMF treatment could improve the myocardial ischaemia/reperfusion (I/R) injury and uncovered its underlying mechanisms.

In our study, we demonstrated for the first time that extracorporeal PEMF has a novel effect on myocardial I/R injury. The number and function of circulating endothelial progenitor cells (EPCs) were increased in PEMF treating rats. The *in vivo* results showed that per-treatment of PEMF could significantly improve the cardiac function in I/R injury group. In addition, PEMF treatment also reduced the apoptosis of myocardial cells by up-regulating the expression of anti-apoptosis protein B-cell lymphoma 2 (Bcl-2) and down-regulating the expression of pro-apoptosis protein (Bax). *In vitro*, the results showed that PEMF treatment could significantly reduce the apoptosis and reactive oxygen species (ROS) levels in primary neonatal rat cardiac ventricular myocytes (NRCMs) induced by hypoxia/reoxygenation (H/R). In particular, PEMF increased the phosphorylation of protein kinase B (Akt) and endothelial nitric oxide synthase (eNOS), which might be closely related to attenuated cell apoptosis by increasing the releasing of nitric oxide (NO). Therefore, our data indicated that PEMF could be a potential candidate for I/R injury.

## Abstract (precis)

Pulsed electromagnetic field (PEMF) treatment protected ischaemia/reperfusion (I/R) injury from apoptosis via B-cell lymphoma 2 (Bcl-2), Bax and nitric oxide (NO) releasing.

## INTRODUCTION

Hypertension, arrhythmia, myocardial infarction (MI) and myocardial ischaemia/reperfusion (I/R) injury are all the most common cardiac diseases, which are the major causes of mortality in the world [1]. Among them, myocardial I/R injury is the most important cause of cardiac damage. Its pathological process is closely related to postoperative complications [2,3] caused by coronary artery vascular formation, coronary revascularization and heart transplantation. After myocardium suffered severe ischaemia, restoration of the blood flow is a prerequisite for myocardial salvage [2]. However, reperfusion may induce oxidative stress [4], inflammatory cell infiltration and calcium dysregulation [5]. All these players contribute to the heart damage such as contraction and arrhythmias [6], generally named myocardial I/R injury. Recently, more and more evolving therapies have been put into use for I/R injury.

Pulsed electromagnetic field (PEMF) is the most widely tested and investigated technique in the various forms of electromagnetic stimulations for wound healing [7], alleviating traumatic pain and neuronal regeneration [8,9]. The rats were randomly divided into PEMF-treated (5 mT, 25 Hz, 1 h daily) and control groups. They hypothesized the possible mechanism that PEMF would increase the myofibroblast population, contributing to wound closure during diabetic wound healing. It is a non-invasive and non-pharmacological intervention therapy. Recent studies indicated that PEMF also stimulated angiogenesis in patients with diabetes [10], and could improve arrhythmia, hypertension and MI [1]. The MI rats were exposed to active PEMF for 4 cycles per day (8 min/cycle, 30±3 Hz, 6 mT) after MI induction. *In vitro*, PEMF induced the degree of human umbilical venous endothelial cells tubulization and increased soluble pro-angiogenic factor secretion [VEGF and nitric oxide (NO)] [7]. However, the role of PEMF in ischaemia and reperfusion diseases remains largely unknown. Our study aimed to investigate the effects of PEMF preconditioning on myocardial I/R injury and to investigate the involved mechanisms.

In our study, we verified the cardioprotective effects of PEMF in myocardial I/R rats and the anti-apoptotic effects of PEMF in neonatal rat cardiac ventricular myocytes (NRCMs) subjected to hypoxia/reoxygenation (H/R). We hypothesized that PEMF treatment could alleviate myocardial I/R injury through elevating the protein expression of B-cell lymphoma 2 (Bcl-2), phosphorylation of protein kinase B (Akt). Meanwhile, it could decrease Bax. We emphatically made an effort to investigate the MI/R model and tried to uncover the underlying mechanisms.

## MATERIALS AND METHODS

### Animals

Male, 12-week-old Sprague Dawley (SD) rats (250–300 g) were purchased from Shanghai SLAC Laboratory Animal. Animals were housed in an environmentally controlled breeding room and given free access to food and water supplies. All animals were handled according to the “Guide for the Care and Use of Laboratory Animals” published by the US National Institutes of Health (NIH). Experimental procedures were managed according to the Institutional Aminal Care and Use Committee (IACUC), School of Pharmacy, Fudan University.

### The measurement of blood pressure in SHR rats

At the end of 1 week treatment with PEMF, the rats were anesthetized with chloral hydrate (350 mg/kg, i.p.), the right common carotid artery (CCA) was cannulated with polyethylene tubing for recording of the left ventricle pressures (MFlab 200, AMP 20130830, Image analysis system of physiology and pathology of Fudan University, Shanghai, China).

### Myocardial I/R injury rat model and measurement of infarct size

All the rats were divided into three groups: (1) Sham: The silk was put under the left anterior descending (LAD) without ligation; (2) I/R: Hearts were subjected to ischaemia for 45 min and then reperfusion for 4 h; (3) I/R + PEMF: PEMF device was provided by Biomobie Regenerative Medicine Technology. The I/R rats were pre-exposed to active PEMF for 2 cycles per day (8 min per cycle), whereas other two groups were housed with inactive PEMF generator. I/R was performed by temporary ligation of the LAD coronary artery for 45 min through an incision in the fourth intercostal space under anaesthesia [11]. Then, the ligature was removed after 45 min of ischaemia, and the myocardium was reperfused for 4 h. Ischaemia and reperfusion were confirmed and monitored by electrocardiogram (ECG) observation. The suture was then tightened again, and rats were intravenously injected with 2% Evans Blue (Sigma–Aldrich). After explantation of the hearts, the left ventricles were isolated, divided into 1 mm slices, and subsequently incubated in 2% 2,3,5-triphenyltetrazolium chloride (TTC; Sigma–Aldrich) in 0.9% saline at 37°C for 25 min, to distinguish infarcted tissue from viable myocardium. These slices were flushed with saline and then fixed in 10% paraformaldehyde in PBS (pH 7.4) for 2 h. Next, the slices were placed on a glass slice and photographed by digital camera, the ImageJ software (NIH) was used in a blind fashion for analysis. Infarct size was expressed as a ratio of the infarct area and the area at risk [12].

### Pulsed electromagnetic field treatment

PEMF were generated by a commercially available healing device (length × width × height: 7 cm × 5cm × 3cm) purchased from Biomoble Regenerative Medicine Technology. The adapter input voltage parameter is approximately 100–240 V and output parameter is 5 V. Fields were asymmetric and consisted of 4.5 ms pulses at 30±3 Hz, with an adjustable magnetic field strength range (*X*-axis 0.22±0.05 mT, *Y*-axis 0.20±0.05 mT, *Z*-axis 0.06±0.02 mT). The I/R rats were housed in custom designed cages and exposed to active PEMF for 2 cycles per time (8 min for 1 cycle), whereas the I/R rats were housed in identical cages with inactive PEMF generator. For *in vitro* study, culture dishes were directly exposed to PEMF for 1–2 cycles as indicated (8 min for 1 cycle, 30 Hz, *X*-axis 0.22 mT, *Y*-axis 0.20 mT, *Z*-axis 0.06 mT) [1]. The background magnetic field in the room area of exposure animals/samples and controls is 0 mT.

### Detection of myocardium apoptosis

Terminal deoxynucleotidyl transferase-mediated dUTP nick-end labelling (TUNEL) assay was applied to analyse cardiomyocyte apoptosis. Heart samples were first fixed in 10% formalin and then paraffin embedded at day 14. Then, the hearts were cut into 5 μm sections. TUNEL staining was carried out as described previously [12]. When apoptosis occurred, cells would look green.

### Determination of myocardial enzymes in plasma

Blood samples were collected after haemodynamic measurement and centrifuged at 3000 ***g*** for 15 min to get the plasma. Creatine kinase (CK), lactate dehydrogenase (LDH), creatine kinase isoenzyme-MB (CKMB) and α-hydroxybutyrate dehydrogenase (HBDH) were quantified by automatic biochemical analyzer (Cobas 6000, Roche). All procedures were performed according to the manufacturer's protocols.

### Myocardium cells morphology via TEM

At the end of the experiment, sections from myocardial samples of left ventricular were immediately fixed overnight in glutaraldehyde solution at 4°C and then incubated while protected from light in 1% osmium tetroxide for 2 h. After washing with distilled water for three times (5 min each), specimens were incubated in 2% uranyl acetate for 2 h at room temperature and then dehydrated in graded ethanol concentrations. Finally, sections were embedded in molds with fresh resin. The changes in morphology and ultrastructure of the myocardial tissues were observed and photographed under a TEM [13].

### Scal-1^+^/flk-1^+^ cells counting of endothelial progenitor cells

We applied antibodies to the stem cell antigen-1 (Sca-1) and fetal liver kinase-1 (flk-1) to sign endothelial progenitor cells (EPCs) as described before, and used the isotype specific conjugated anti-IgG as a negative control. The amount of Scal-1^+^/flk-1^+^ cells would be counted by flow cytometry technique [14].

### Measurement of nitric oxide concentration and Western blotting

Plasma concentrations of NO were measured with Griess assay kit (Beyotime Institute of Biotechnology) according to the manufacturer's protocol. The expressions of Bax, Bcl-2, p-Akt, Akt, p-endothelial nitric oxide synthase (eNOS), eNOS and glyceraldehyde-3-phosphate dehydrogenase (GAPDH) were assessed using Western blot as described recently [15]. Proteins were measured with Pierce BCA Protein Assay Kit (Thermo). Hippocampal protein lysates (50 mg/well) were separated using (SDS/PAGE) under reducing conditions. Following electrophoresis, the separated proteins were transferred to a PVDF membrane (Millipore). Subsequently, non-specific proteins were blocked using blocking buffer (5% skim milk or 5% BSA in T-TBS containing 0.05% Tween 20), followed by overnight incubation with primary rabbit anti-rat antibodies specific for target proteins as mentioned before (Cell Signaling Technology) at 4°C. Blots were rinsed three times (5 min each) with T-TBS and incubated with horseradish peroxidase (HRP)-conjugated secondary antibody (1:10000, Proteintech) for 2 h at room temperature. The blots were visualized by using enhanced chemiluminescence (ECL) method (Thermo). GAPDH was applied to be the internal control protein. Intensity of the tested protein bands was quantified by densitometry.

### Cell culture

Primary neonatal rat cardiac ventricular myocytes (NRCMs) were collected as previously described [15]. Briefly, the ventricles of new born SD rats (1–3 days old) were minced and digested with 0.125% trypsin. Isolated cardiomyocytes were cultured in Dulbecco's modified Eagle's medium/F-12 (DMEM/F12, Life Technologies) supplemented with 10% (v/v) FBS (Life Technologies), 100 units/ml penicillin and 100 mg/ml streptomycin. The following experiments used spontaneously beating cardiomyocytes 48–72 hours after plating. (37°C with 5% CO_2_).

### Cell treatment (hypoxia/reoxygenation)

NRCMs were prepared according to the methods recently described [15]. To establish the H/R model, the cells were cultured in DMEM/F-12 without glucose and serum. The cells were exposed to hypoxia (99% N_2_+5% CO_2_) for 8 h, followed by reoxygenation for 16 h. The cells were pretreated with PEMF for 30 min before the H/R procedure. The control group was cultured in DMEM/F-12 with low glucose (1000 mg/l) and 2% serum under normoxic air conditions for the corresponding times.

### Cell viability assays

The viability of NRCMs cultured in 96-well plates was measured by using the Cell Counting Kit-8 (CCK-8) (Dojindo Molecular Technologies) according to the manufacturer's instructions. The absorbance of CCK-8 was obtained with a microplate reader at 450 nm.

### Measurement of intracellular reactive oxygen species levels

Reactive oxygen species (ROS) levels in NRVMs were determined by dihydroethidium (DHE, Sigma–Aldrich) fluorescence using confocal microscopy (Zeiss, LSM 710). After different treatments, cells were washed with D-PBS and incubated with DHE (10 μmol/l) at 37°C for 30 min in the dark. Then, residual DHE was removed by PBS-washing. Fluorescent signals were observed (excitation, 488 nm; emission, 610 nm) under a laser confocal microscope (Zeiss).

### Data analysis

All the data were presented as means ± S.E.M. Differences were compared by one-way ANOVA analysis by using SPSS software version 19.0 (SPSS) and *P* value <0.05 was taken as statistically significant.

## RESULTS

### PEMF could lower blood pressure under treatment of certain PEMF intensity in SHR rat model (double-blind)

**Figure 1:**
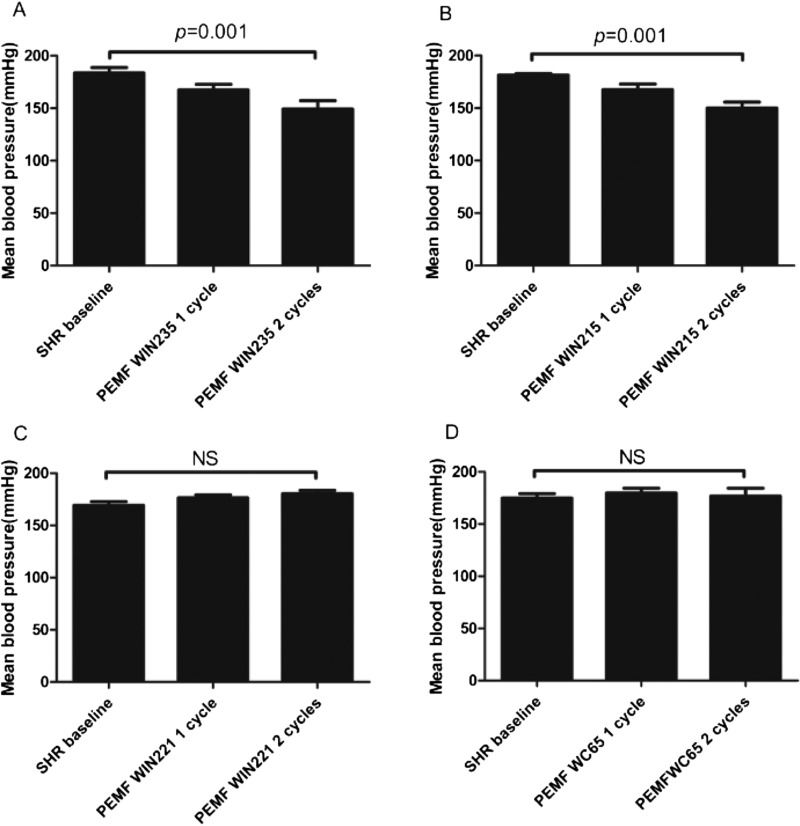
The effect of PEMF on SHR rats *in vivo.* PEMF could lower the blood pressure in SHR rats. At day 7 treatment with different intensity PEMF, blood pressure was recorded via CCA [1(**A**), 1(**B**), 1(**C**) and 1(**D**)]. Data were represented as the mean ± S.E.M. Differences were compared by one-way ANOVA analysis by using SPSS software version 19.0 (SPSS) and *P* value <0.05 was taken as statistically significant; (*n*=8–10 in each group).

To determine whether PEMF has any effects on blood pressure of SHR rats, we treated SHR rats with different PEMF intensity 1–4 cycles per day for 7 days and measured the blood pressure changes via CCA. We observed that PEMF treatment could significantly lower the blood pressure in the Bioboosti WIN235 and WI215-stimulating groups than that in non-treated ones (Figures 1A and 1B). But Bioboosti WIN221 and WC65 treating groups did not have any effects on the blood pressure in SHR rats, compared with the non-treated ones (Figures 1C and 1D). Fields were asymmetric and consisted of 4.5 ms pulses at 30±3 Hz, with an adjustable magnetic field strength range (*X*-axis 0.22±0.05 mT, *Y*-axis 0.20±0.05 mT, *Z*-axis 0.06±0.02 mT). The I/R rats were housed in custom designed cages and exposed to active PEMF for 2 cycles per time (8 min for 1 cycle), whereas the I/R rats were housed in identical cages with inactive PEMF generator.

According to this result, we chose Bioboosti WIN235 as our needed PEMF to carry out the following experiments.

### PEMF treatment could observably improve the abundance of EPCs

**Figure 2:**
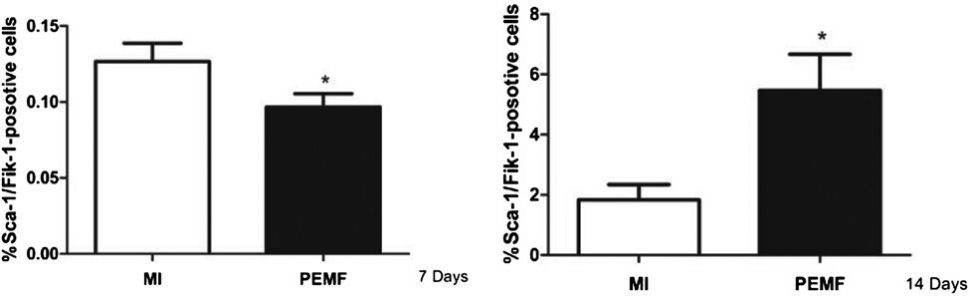
The effect of PEMF on the number of Scal-1^+^/flk-1^+^ cells after treating EPSc for 7 and 14 days. PEMF treatment notably increased the number of Scal-1^+^/flk-1^+^ cells after treating EPSc for 7 and 14 days. Data were represented as the mean ± S.E.M. Differences were compared by one-way ANOVA analysis by using SPSS software version 19.0 (SPSS) and *P* value <0.05 was taken as statistically significant; (*n*=10 in each group).

Amplifying EPCs abundance and function is an active focus of research on EPCs-mediated neovascularization after I/R. Thus, the number of circulating EPCs was identified by Sca-1/flk-1 dual positive cells as described. We determined that PEMF treatment could remarkably increase the number of Scal-1^+^/flk-1^+^ cells in peripheral blood at postoperative days 7 and 14 (Figure 2).

### Preliminary assessment of PEMF showed great protective effect against myocardial infarction/reperfusion injury (MI/RI) rat model

**Figure 3:**
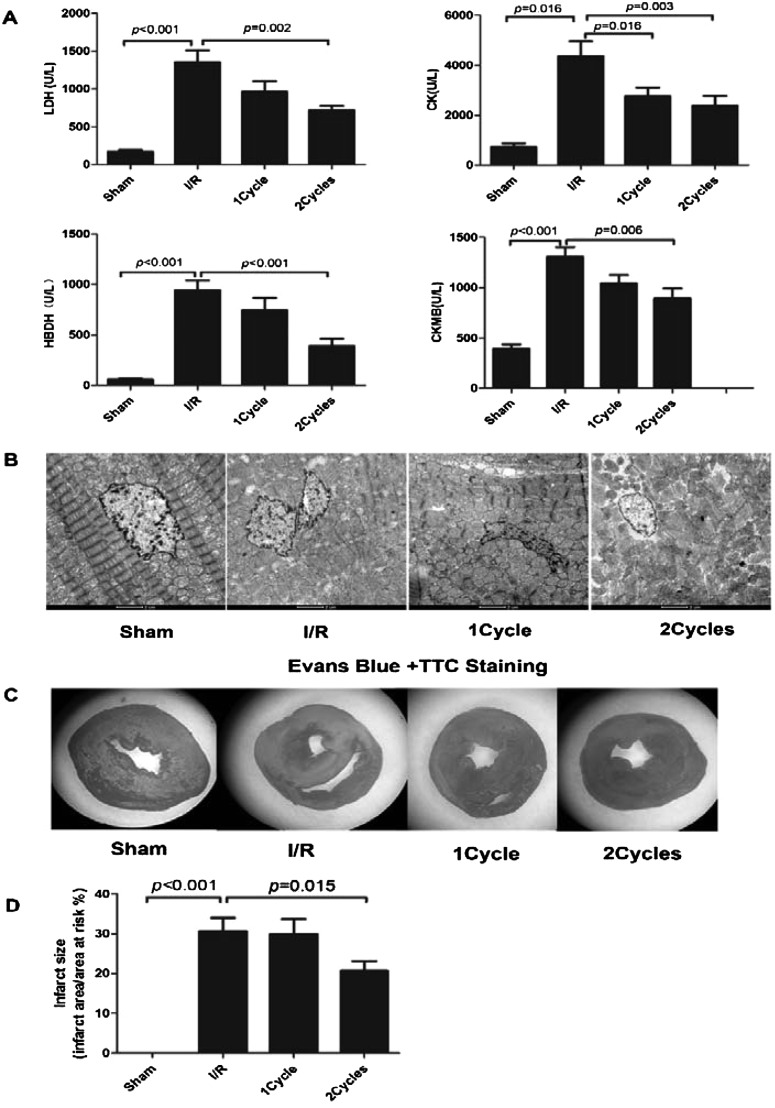
Protective effect of PEMF on I/R rats *in vivo*. Plasma myocardial enzymes (LDH, CK, HBDH and CKMB) content was quantified by automatic biochemical analyzer (**A**) (*n*=18 in each group). Changes on cardiac cell morphology via TEM (**B**) (*n*=6 in each group). TTC-Evans Blue staining for MI area (**C**). Statistical analysis of the effect of PEMF in reducing infarct size in a rat model of I/R (D). Data were represented as the mean ± S.E.M. Differences were compared by one-way ANOVA analysis by using SPSS software version 19.0 (SPSS) and *P* value <0.05 was taken as statistically significant.

To examine the effect of PEMF on myocardial I/R, male SD rats were divided into three groups: Sham, I/R and I/R+ PEMF (2 cycles per day, 8 min per cycle) per day until 28 days. We observed that PEMF stimulation could significantly decrease four plasma myocardial enzymes (LDH, CK, CKMB and HBDH) in I/R rats (Figure 3A). Additionally, we found that pre-stimulating PEMF could improve the cardiac morphology via TEM, compared with I/R+ PEMF group. TEM revealed the rupture of muscular fibres, together with mitochondrial swelling, and intracellular oedema in Group I/R. The shape of nucleus was irregular, with evidence of mitochondrial overflow after cell death. Compared with Group I/R+ PEMF, less muscular fibres were ruptured, with mild swelling of mitochondria, mild intercellular oedema and less cell death. In Group Sham, the ruptured muscular fibres, mitochondrial or intracellular oedema and dead cells were not observed (Figure 3B). To further confirm protective effect of PEMF, we measured the MI size by applying TTC and Evans Blue staining in all three groups. The MI area in I/R+ PEMF group could be reduced, compared with the model rats in I/R group (Figure 3C).

### *In vivo*, PEMF dramatically reduced cell apoptosis induced by I/R injury

**Figure 4:**
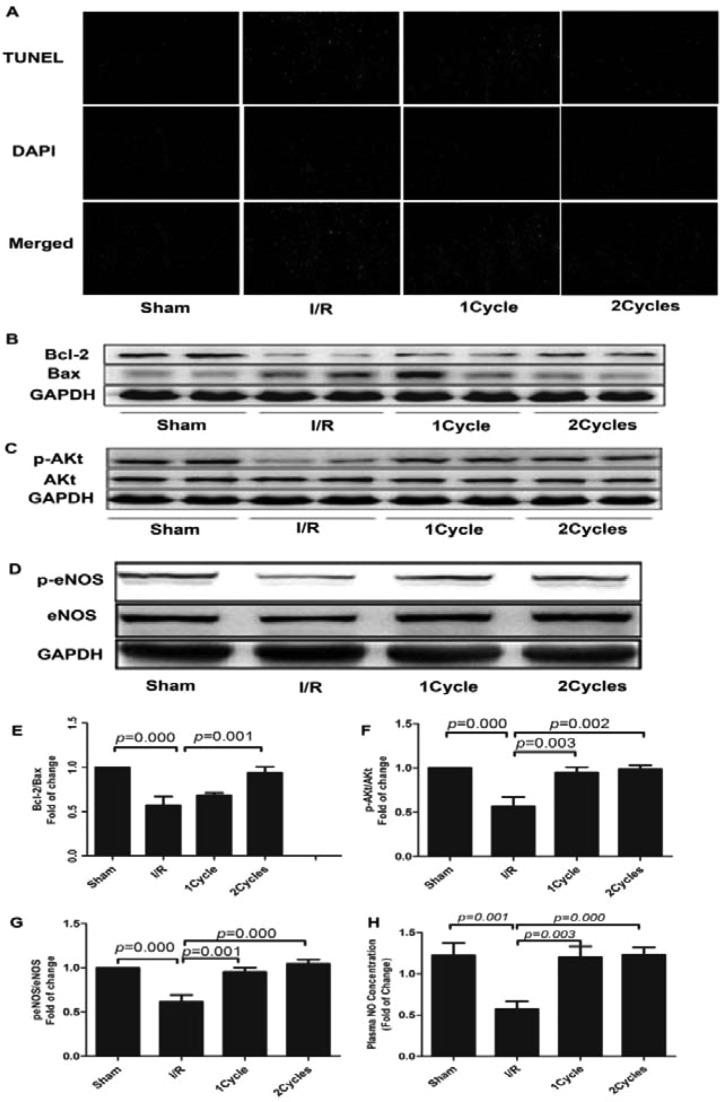
Apoptotic cardiomyocyte was identified by TUNEL analysis, apoptotic cardiomyocyte appears green whereas TUNEL-negative appears blue (**A**), photomicrographs were taken at ×200 magnification. Apoptosis-related protein Bcl-2, Bax, p-Akt level of different treatments, p-eNOS level of different treatments, which were measured by Western blot analysis (**B**–**G**). Plasma concentrations of NO were measured by spectrophotometer assay (**H**) (*n*=8 in each group). Data were represented as the mean ± S.E.M. Differences were compared by one-way ANOVA analysis by using SPSS software version 19.0 (SPSS); *P*<0.05, compared with the MI/RI ones (*n*=8 in each group).

As H/R of cardiomyocytes contributed to cell death, we also detected the effect on myocardial apoptosis by using TUNEL kit, as shown in Figure 4(A). We uncovered that PEMF pretreating could dramatically decrease apoptosis of myocardial cells in I/R + PEMF group, compared with I/R group. In addition, we also found that PEMF treatment could significantly increase the expression of anti-apoptosis protein Bcl-2, p-eNOS and p-Akt and down-regulated the expression of pro-apoptosis protein Bax in the heart tissue, as shown in Figure 4(B).

### The effect of PEMF on cell viability in neonatal rat cardiac ventricular myocytes

**Figure 5:**
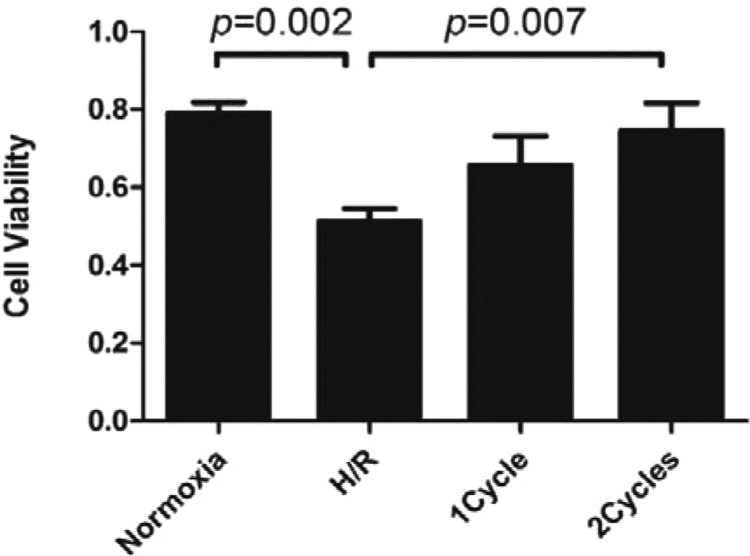
NRCMs viability measured by CCK-8 assay at the end of the treatment for 72 h. PEMF treatment enhanced the cell viability of hypoxia NRCMs. Data were represented as the mean ± S.E.M. Differences were compared by one-way ANOVA analysis by using SPSS software version 19.0 (SPSS) and *P* value <0.05 was taken as statistically significant; *P*<0.05, compared with the H/R ones (*n*=17 in each group).

To further investigate whether PEMF has the same effect *in vitro*, we simulated the I/R injury model *in vitro*. We applied NRCMs and hypoxia incubator to mimic myocardial I/R injury via H/R as described in the section ‘Materials and Methods’. We found that PEMF treatment (2 cycles) could remarkably improve cell viability, compared with the H/R group (Figure 5). For *in vitro* study, culture dishes were directly exposed to PEMF for 1–2 cycles as indicated (8 min for 1 cycle, 30±3 Hz, *X*-axis 0.22±0.05 mT, *Y*-axis 0.20±0.05 mT, *Z*-axis 0.06±0.02 mT).

### Specific-density PEMF could decrease intracellular ROS levels of primary cardiomyocytes subjected to hypoxia/reperfusion

**Figure 6:**
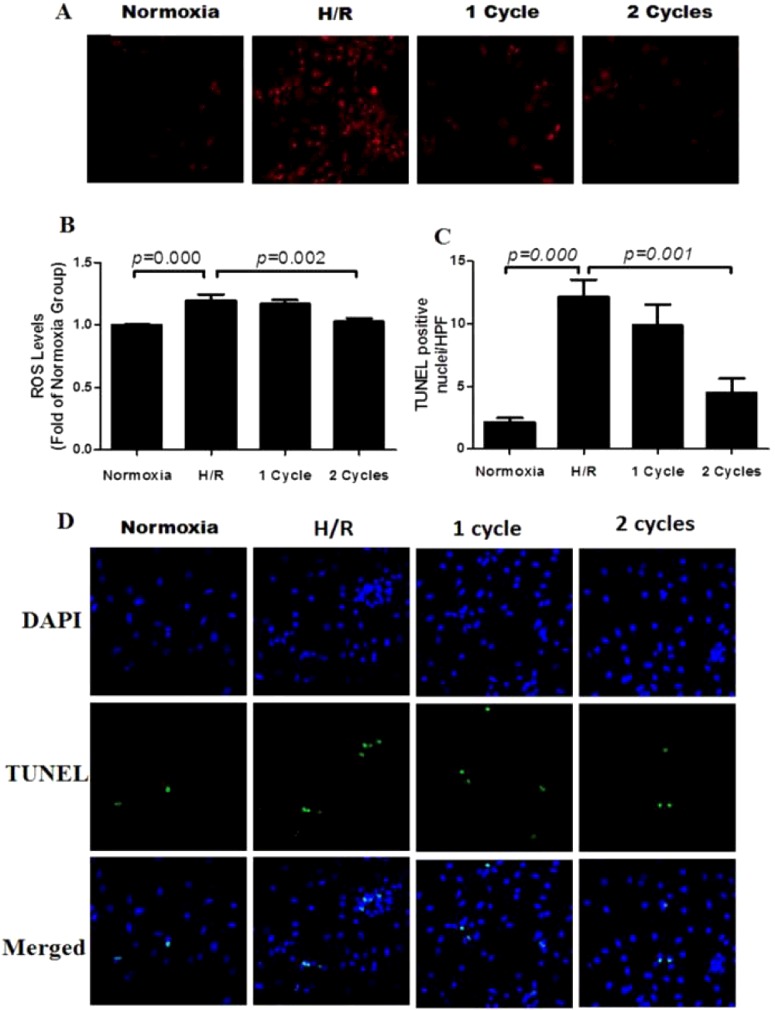
PEMF protected Neonatal rat cardiac ventricular myocytes (NRCMs) from hypoxia/reoxygenation (H/R)-induced apoptosis via decreasing ROS levelat the end of the treatment for 72 h *in vitro*. Effect of PEMF on ROS levels and apoptosis induced by hypoxia/reoxygenation *in vitro.* (**A**) Representative images of ROS levels in NRCMs detected by confocal microscope. (**B**) Quantitative analysis for ROS levels in NRCMs were detected by microplate reader. (**C**) TUNEL-positive nuclei quantification represented as number per high-power field (HPF). (**D**) Representative photographs of cardiomyocyte apoptosis from NRCMs detected by confocal microscope-TUNEL (green), apoptotic nuclei, DAPI (blue) and total nuclei. Data were represented as the mean ± S.E.M. Differences were compared by one-way ANOVA analysis by using SPSS software version 19.0 (SPSS) and *P* value <0.05 was taken as statistically significant, (*n*=6 in each group).

As shown in Figure 6(A), NRCMs that were subjected to H/R increased significantly the ROS level, whereas the ROS level had been decreased in PEMF group (2 cycles), in contrast with the H/R group. Representative images of the ROS level were displayed in Figure 6(B). At the same time, we identified the effect on NRCMs apoptosis after suffering H/R by using TUNEL kit. As shown in Figure 6(C), cell apoptosis in the H/R group was aggravated, whereas PEMF treatment could reduce the cell death. Representative images of TUNEL staining were shown in Figure 6(D).

### Effect of PEMF on NO releasing via Akt/eNOS pathway

**Figure 7:**
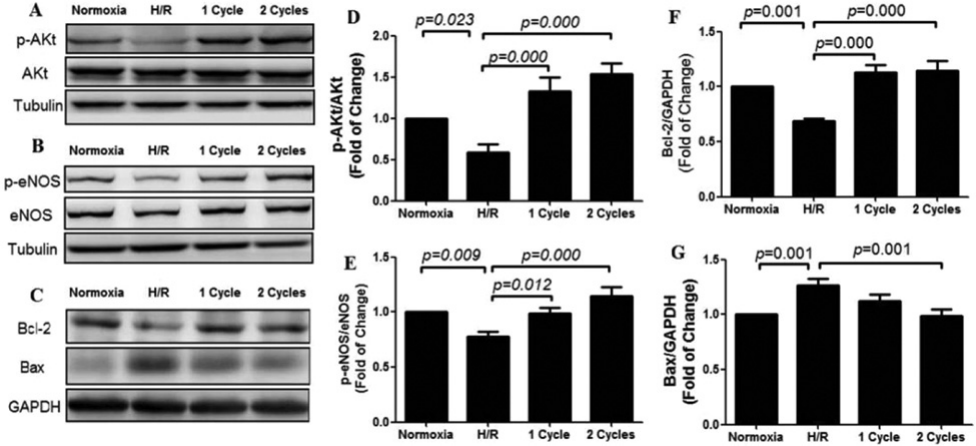
The related protein expression about the effect of PEMF on apoptosis induced by hypoxia/reoxygenationat the end of the treatment for 72 h *in vitro*. PEMF increased the phosphorylation of Akt, endothelial nitric oxide synthase (eNOS), and the expression of Bcl-2 anddown-regulated the expression of Bax. The related protein expression about the effect of PEMF on apoptosis induced by H/R *in vitro.* PEMF could increase NO releasing. (**A**, **D**) p-AKt level of different treatments. (**B**, **E**) p-eNOS level of different treatments. (**C**, **F**, **G**) Apoptosis-related proteins Bcl-2, Bax, which were measured by Western blot analysis. Data were represented as the mean ± S.E.M. Differences were compared by one-way ANOVA analysis by using SPSS software version 19.0 (SPSS) and *P* value <0.05 was taken as statistically significant, (*n*=8 in each group).

Cultured NRCMs were treated with PEMF stimulation for 1 to 2 cycles and the supernatant and cell lysate were collected. When cells suffered H/R, intracellular levels of p-Akt, p-eNOS and Bcl-2 were decreased, whereas PEMF treatment could increase the phosphorylation of Akt, p-eNOS and Bcl-2 (Figures 7A–7C). The expression of Bax was increased when cells subjected to H/R whereas PEMF treatment reversed such increase (Figure 7C). Western blot analysis was shown in Figure 7(D) for p-Akt/Akt, Figure 7(E) for p-eNOS/eNOS, Figure 7(F) for Bcl-2 and Figure 7(G) for Bax.

## DISCUSSION

Our present study provides the first evidence that PEMF has novel functions as follows: (1) We treated SHR rats with different PEMF intensity (8 min for 1 cycle, 30±3 Hz, *X*-axis 0.22±0.05 mT, *Y*-axis 0.20±0.05 mT, *Z*-axis 0.06±0.02 mT) 1–4 cycles per day for 7 days. PEMF can lower blood pressure under treatment of certain PEMF intensity in SHR rat model (double-blind). (2) PEMF has a profound effect on improving cardiac function in I/R rat model. (3) PEMF plays a vital role in inhibiting cardiac apoptosis via Bcl-2 up-regulation and Bax down-regulation. (4) *In vitro*, PEMF treatment also has a good effect on reducing ROS levels by Akt/eNOS pathway to release NO and improving cell apoptosis in NRCMs subjected to hypoxia.

Many previous studies showed that extracorporeal PEMF-treated(5 mT, 25 Hz, 1 h daily) could enhance osteanagenesis, skin rapture healing and neuronal regeneration, suggesting its regenerative potency [8,16,17]. And some researchers had found that PEMF therapy (8 min/cycle, 30±3 Hz, 6 mT) could improve the myocardial infarct by activating VEGF–Enos [18] system and promoting EPCs mobilized to the ischaemic myocardium [1,19]. Consistent with the previous work, our present study demonstrated that PEMF therapy could significantly alleviate cardiac dysfunction in I/R rat model.

Recent evidence suggest that circulating EPCs can be mobilized endogenously in response to tissue ischaemia or exogenously by cytokine stimulation and the recruitment of EPCs contributes to the adult blood vessels formation [19,20,21]. We hypothesized that PEMF could recruit more EPCs to the vessels. To confirm our hypothesis, we applied antibodies to the Sca-1 and flk-1 to sign EPC. The results indicated that PEMF could remarkably increase the number of EPCs in the PEMF group, compared with the I/R group.

Previous evidence indicated that when heart suffered I/R, cardiac apoptosis would be dramatically aggravated [22–24]. Myocardial apoptosis plays a significant role in the pathogenesis of myocardial I/R injury. We assumed that PEMF might play its role in improving cardiac function through inhibiting cell apoptosis. The Bcl-2 family is a group of important apoptosis-regulating proteins that is expressed on the mitochondrial outer membrane, endoplasmic reticulum membrane and nuclear membrane. Overexpression of Bcl-2 proteins blocks the pro-apoptosis signal transduction pathway, thereby preventing apoptosis caused by the caspase cascade [25]. The role Bax plays in autophagy is a debatable. Recently, new genetic and biochemical evidence suggest that Bcl-2/Bcl-xL may affect apoptosis through its inhibition of Bax [26]. Overexpression of Bax protein promotes the apoptosis signal pathway. In the present study, we applied TUNEL staining to find that PEMF has a perfect effect on cardiac cell apoptosis by regulating apoptosis-related proteins Bcl-2 and Bax [25,26,27,28].

To verify our findings in the rat model, we mimicked I/R condition *in vitro* by hypoxia exposure in NRCMs. Results showed that not only *in vivo*, hypoxia could induce cell apoptosis *in vitro*. And we also found that PEMF treatment could significantly alleviate cell apoptosis induced by hypoxia. At the basal level, ROS play an important role in mediating multiple cellular signalling cascades including cell growth and stress adaptation. Conversely, excess ROS can damage tissues by oxidizing important cellular components such as proteins, lipids and DNA, as well as activating proteolytic enzymes such as matrix metalloproteinases [29]. Previous studies showed that when cells were subjected to hypoxia, the intracellular ROS level would be sharply increased, and the overproduction of ROS would result in cell damage [19,30,31]. In the present study, PEMF treatment could prominently down-regulate ROS levels. We also investigated how PEMF reduced the intracellular ROS level.

NO appears to mediate distinct pathways in response to oxidative stress via AKt–eNOS pathway [32,33]. NO is identified as gaseous transmitters. In vascular tissue, NO is synthesized from L-arginine by nitric oxide synthase (NOS) and it is considered to be the endothelium-derived relaxing factor. Evidence show that the NO generation in endothelium cells was damaged in hypertensive patients [34]. NO could also prevent platelet activation and promote vascular smooth muscle cells proliferation [35]. NO generation from eNOS is considered to be endothelium-derived relaxing and ROS-related factor [36,37]. Some researchers found that bradykinin limited MI induced by I/R injury via Akt/eNOS signalling pathway in mouse heart [38]. And bradykinin inhibited oxidative stress-induced cardiomyocytes senescence by acting through BK B2 receptor induced NO release [39]. Such evidence indicated that Akt phosphorylation could activate eNOS, which lead to NO releasing, and resulted in ROS reducing. In the present study, we found that PEMF decreased ROS via Akt/eNOS pathway.

In conclusion, this is the first study suggesting that PEMF treatment could improve cardiac dysfunction through inhibiting cell apoptosis. Furthermore, *in vitro*, we first clarified PEMF still plays a profound effect on improving cell death and removing excess ROS via regulating apoptosis-related proteins and Akt/eNOS pathway. All these findings highlight that PEMF would be applied as a potentially powerful therapy for I/R injury cure.

## Abbreviations

Akt: protein kinase B
Bax: Bcl-2 associated X protein
Bcl-2: B-cell lymphoma 2
CCA: common carotid artery
CCK-8: Cell Counting Kit-8
CK: creatine kinase
CKMB: creatine kinase isoenzyme-MB
DAPI: 4,6′-diamidino-2′-phenylindole
DHE: dihydroethidium
DMEM/F12: Dulbecco's modified Eagle's medium/F-12
dUTP: deoxyuridine triphosphate
eNOS: endothelial nitric oxide synthase
EPCs: endothelial progenitor cells
flk-1: fetal liver kinase-1
GAPDH: glyceraldehyde-3-phosphate dehydrogenase
HBDH: α-hydroxybutyrate dehydrogenase
H/R: hypoxia/reoxygenation
HRP: horseradish peroxidase
I/R: ischaemia/reperfusion
LAD: left anterior descending
LDH: lactate dehydrogenase
MI: myocardial infarction
MI/R: myocardial infarction/reperfusion
MI/RI: myocardial infarction/reperfusion injury
NRCMs: neonatal rat cardiac ventricular myocytes
PEMF: pulsed electromagnetic field
ROS: reactive oxygen species
Sca-1: stem cell antigen-1
SD: Sprague Dawley
SHR: spontaneously hypertensive rats
TTC: 2,3,5-triphenyltetrazolium chloride
TUNEL: terminal deoxynucleotidyl transferase-mediated dUTP nick-end labelling
VEGF: vascular endothelial growth factor
